# Rationale and design of the INNOVATE Trial: an international cooperative study on surgical versus conservative treatment for odontoid fractures in the elderly

**DOI:** 10.1186/1471-2474-15-7

**Published:** 2014-01-08

**Authors:** Jeroen GJ Huybregts, Wilco CH Jacobs, Wilco C Peul, Carmen LA Vleggeert-Lankamp

**Affiliations:** 1 Department of Neurosurgery, Leiden University Medical Centre, PO Box 9600, 2300 RC Leiden, The Netherlands

**Keywords:** Odontoid fractures, Elderly, Surgical treatment, Conservative treatment, Prospective cohort study, Comparative, Observational, Cervical spine, Octogenarians, Nonagenarians

## Abstract

**Background:**

Fractures of the odontoid process of the axis are the most common fractures of the geriatric cervical spine. As the population ages, their incidence is expected to increase progressively, as is the number of very old patients (>80 years) with an odontoid fracture. No consensus exists on the optimal treatment (surgical or conservative) and the most relevant outcome parameter (osseous union, fracture stability or clinical outcome). The aim of the INNOVATE (INterNational study on Odontoid frActure Treatment in the Elderly) Trial is to prospectively assess fracture healing and clinical outcome after surgical and conservative treatment for odontoid fractures in the elderly patient, with a specific focus on the very old patient.

**Methods/Design:**

The trial is an observational study in which eleven centres in five European countries are involved. All patients admitted to one of these centres who meet the selection criteria (≥55 years, acute (<two weeks) type II/III odontoid fracture, no rheumatoid arthritis, no ankylosing spondylitis, no previous treatment for odontoid fracture) are asked to participate. The applied treatment is in accordance with usual care and chosen by the treating surgeon and patient. A cohort of 275 patients will be included. Clinical and radiological follow-up moments are scheduled at 6, 12, 26, 52 and 104 weeks, at which both surgeon and patient will complete Case Record Forms (CRFs). The primary outcome will be a combination of fracture healing and clinical outcome at 52 weeks. Osseous union and fracture stability will be assessed with CT-imaging and dynamic X-ray. Clinical outcome will be scored by the Neck Disability Index (NDI) and correlated to the imaging data. Additionally, predefined subgroup analysis will be carried out (i.e. for patient age and osteoporosis) and prognostic factors will be identified.

**Discussion:**

Evidence for the optimal treatment for odontoid fractures is lacking. Focusing on both fracture healing and clinical outcome, the results of this study will yield valuable information enabling more rational decision making in the treatment for odontoid fractures in the elderly.

**Trial registration:**

Netherlands Trial Register NTR3630

## Background

In the elderly, odontoid fractures are the most common fractures of the cervical spine [1-6]. As the population ages, their incidence and relevance to clinical practice are expected to increase [7]. The treatment for patients with fractures of the odontoid process is based on the fracture pattern, the patient’s medical condition [2] including age, pain, neurological deficits, and the surgeon’s personal preference. Surgical treatment involves anterior odontoid screw fixation or posterior (cranio)atlanto-axial arthrodesis, leading to prompt stability of the upper cervical spine. However, the condition of the patient may deteriorate by undergoing (major) cervical spine surgery [8]. Especially in the very old (≥80 years of age), a surgical intervention has significant risks for the patient. An alternative to avoid the possible complications of cervical spine surgery is conservative treatment with rigid or non-rigid immobilisation for a longer period of time. However, such immobilisation may eventually result in non-union and prolonged fracture instability, requiring secondary surgery [9]. This unnecessarily lengthens treatment duration and, worse, can cause significant deterioration of the cervical spine anatomy and the patient’s condition [10].

Finding the right balance between fracture healing and treatment complications is difficult. In very old patients, finding this balance is even more challenging. In hospitals where upper cervical spine surgery is frequently performed, it is becoming increasingly common to operate on even the very old patient with a cervical spine fracture. However, debate remains as to whether or not this is indeed a favourable development.

Currently available literature reviews on this topic were inconclusive [11,12]. Recent clinical studies focussed on survival and the occurrence of complications, but not on fracture union and stability [13,14]. All other performed clinical studies were carried out retrospectively, were of limited quality and most did not specifically focus on elderly patients. Moreover, minimal attention was paid to grounds for chosen treatments, and patient groups were often poorly comparable. In the vast majority of published studies, only a small number of patients were included (typically <50).

Furthermore, the goal of treatment is still debatable. It is unknown whether or not non-union always leads to complaints in the patient. Consequently, debate remains as to whether the goal of treatment should be osseous union, fracture stability or a favourable clinical outcome. The measurement of these parameters is likewise not uniformly described. The available literature shows higher osseous union rates in surgically compared to conservatively treated patients (66–85% and 28–44%, respectively), but patient selection mechanisms may have profoundly interfered with these outcome percentages. The majority of patients achieved fracture stability regardless of the applied treatment (82-97% in surgically treated patients and 53-79% in conservatively treated patients). There are insufficient data available, especially from direct comparisons, to determine the difference in clinical outcome between surgical and conservative treatment strategies. There is no evidence that clinical outcome correlates better to fracture union than to fracture stability, or that the quality of union, whether it be osseous of fibrous, influences clinical outcome [12].

The goal of this study is to prospectively compare fracture union, fracture stability and NDI improvement at 52 weeks between surgical and conservative treatments in patients over 55 years of age with acute type II and III odontoid fractures. Predefined subgroup analysis may offer prognostic factors that can predict the success of either a surgical or conservative treatment. The influence of age (≥55-80 and ≥80 years) on treatment outcome will particularly be studied. The outcome of this study will yield valuable information enabling more rational decision making in treating the elderly patient population.

## Design and methods

**Figure 1:**
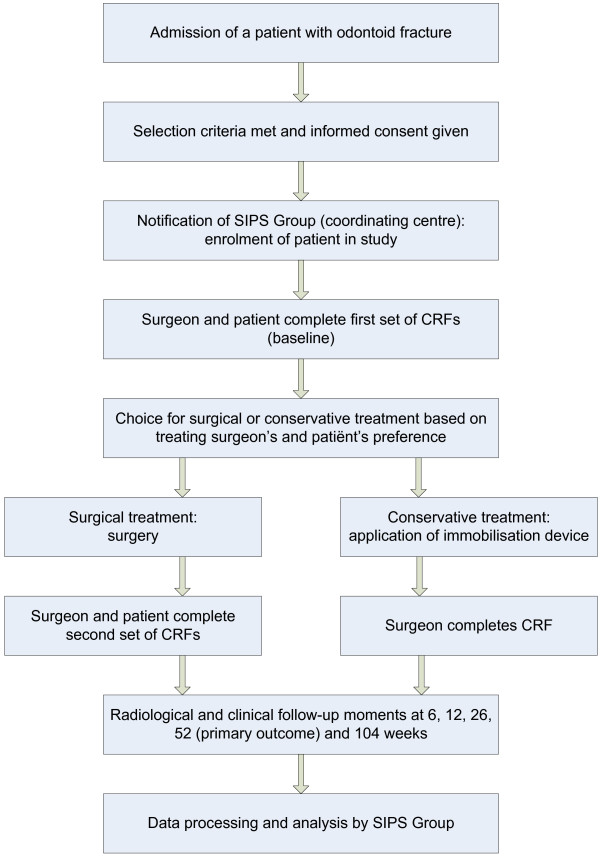
Flow chart of the INNOVATE Trial.

The INNOVATE (INterNational study on Odontoid frActure Treatment in the Elderly) Trial is a prospective, comparative cohort study with two parallel groups. A multi-centre study is necessary to include the required number of patients in a favourable time frame and to obtain generalisable results. The trial will be conducted in eleven hospitals in five European countries (Table 1). Medical ethical approval was obtained in all participating centres prior to the start of the study. In a number of other hospitals, the trial is still pending approval after which these centres will also participate. The participating centres are individually responsible for the treatment applied. The coordination of the study will be carried out by the Spine Intervention Prognostic Study (SIPS) Group of the Leiden University Medical Centre. Experienced in conducting multi-centre national and international studies, the SIPS Group has established research databases and has a group of research nurses available. The research nurses will monitor data collection and can be consulted by surgeons and patients who have questions about the study protocol or the treatment of individual subjects. The main research question will be answered at 52 weeks of follow-up. The complete follow-up period will be 104 weeks (Figure 1).

**Table 1:** Participating medical centres

	**Centre**	**Department**	**Local investigator(s)**	**Medical ethics committee**
**Austria**	Universitäres Lehrkrankenhaus Feldkirch, Feldkirch	Traumasurgery	Osti	Not applicable*
**Belgium**	University Hospital Leuven, Leuven	Neurosurgery	Depreitere	Commissie Medische Ethiek U.Z. K.U. Leuven
**Italy**	Catholic University Rome, Rome	Neurosurgery	Visocchi	Not applicable*
**The Netherlands**	Leiden University Medical Centre, Leiden (coordinating centre)	Neurosurgery	Vleggeert-Lankamp	Commissie Medische Ethiek LUMC
	Medical Centre Haaglanden, The Hague	Neurosurgery	Arts	Commissie Medische Ethiek LUMC
	University Medical Centre Nijmegen, Nijmegen	Neurosurgery	Bartels	Commissie Medische Ethiek LUMC
	University Medical Centre Groningen, Groningen	Neurosurgery	Coppes	Commissie Medische Ethiek LUMC
	VU Medical Centre, Amsterdam	Neurosurgery	Noske	Commissie Medische Ethiek LUMC
	Academic Medical Centre, Amsterdam	Neurosurgery	Bouma	Commissie Medische Ethiek LUMC
	University Medical Centre Utrecht, Utrecht	Neurosurgery, Orthopaedics	Slooff, Öner	Commissie Medische Ethiek LUMC
**Spain**	Spine Unit of the Vall d’Hebron University Hospital, Barcelona	Orthopaedics	Pellisé	Comité Ético de Investigación Clínica del Hopital Universitario de Vall d’Hebron

*In these countries, no medical ethical approval is required for observational studies.

### Patient selection

All patients admitted to one of the participating centres who meet the selection criteria will be asked to participate in the study (Table 2). Prior to the start of treatment, the patient will be enrolled by notification to the SIPS Group. The inclusion of patients will continue until the target sample size is reached.

**Table 2:** Selection criteria

**Inclusion criteria**	• At least 55 years old
	• Acute type II and III odontoid fracture based on the classification by Anderson and d’Alonzo (possibly in combination with other fractures); diagnosed using computed tomography
	• Less than two weeks post injury
	• Informed consent
**Exclusion criteria**	• Rheumatoid arthritis
	• Ankylosing spondylitis
	• Previous treatment for odontoid fracture
	• Communication with patient is hampered (e.g. language barrier, severe cognitive impairment, coma)

During baseline and follow-up appointments, radiological and clinical data will be gathered. Patients will also be sent questionnaires to answer at home. Questionnaires will focus on pain intensity, general wellbeing, perceived recovery and illness-related inconveniences.

### Treatment

The treating surgeon and patient will make a shared decision as to whether surgical or conservative treatment will be applied. Participating centres and surgeons are all able to facilitate and respectively carry out both surgical and conservative treatments.

#### Surgical treatment

Surgical treatment can be carried out by either an anterior or posterior approach. In an anterior approach, a single or double odontoid screw is inserted through the corpus of C2 into the odontoid process to directly stabilise the fracture (anterior odontoid screw fixation). In the posterior approach, fixation of the C1-C2 vertebrae is carried out, thereby indirectly immobilising the odontoid process as well (C1-C2 arthrodesis). The posterior technique is sometimes extended cranially to C0 or caudally to C3 or lower, possibly leading to increased stability but further limiting the cervical range of motion. A CRF will register the procedures/findings of the surgeon and the motivation for the chosen surgical treatment strategy.

#### Conservative treatment

Conservative treatment involves a variety of devices by which a patient’s cervical spinal column is rigidly or non-rigidly externally immobilised. Rigid immobilisation is mostly carried out by application of halo-vest traction. Non-rigid immobilisation is achieved by application of a hard cervical collar (e.g. Philadelphia or Miami-J collar). A CRF will register the procedure and motivation for the chosen conservative treatment strategy.

### Baseline assessment

After inclusion and prior to the start of treatment, both surgeon and patient will complete the first set of CRFs. In the case of a surgical treatment, the surgeon will complete a CRF directly after surgery and the patient will complete a second set of CRFs three days after surgery to assess the short-term effect of the operation for the patient. In the case of a conservative treatment, the surgeon will complete a CRF after the application of the immobilisation device.

### Follow-up period

Patients will be seen by the treating surgeon five times for radiological and/or clinical follow-up visits; at 6, 12, 26, 52 and 104 weeks after the start of treatment. In addition to usual patient care, patients will be sent questionnaires (CRFs) to complete at home prior to each follow-up visit (Table 3). On no occasion will patients see the results of earlier assessments. After the last follow-up visit at 104 weeks the patient’s participation in the study ends.

**Table 3:** Data collection and outcome measures

	**Baseline**	**Immediately after start of treatment**	**Weeks of follow-up**				
			**6**	**12**	**26**	**52**	**104**
General status and fracture assessment	**X**						
Demographic data	**X**		**X**	**X**	**X**	**X**	**X**
NDI, MDI, VAS neck pain, SF-36, EQ-5D	**X**	X*	**X**	**X**	**X**	**X**	**X**
DS14, IPQ-K, Likert			**X**	**X**	**X**	**X**	**X**
Complications of surgery		X**	X**	X**	X**	X**	X**
Secondary surgery			X**	X**	X**	X**	X**
Dynamic X-ray					**X**	**X**	**X**
CT-Cervical spine	**X**			**X**	X**	**X**	

*In case of surgical treatment only.

**When indicated.

### Primary outcome

The primary outcomes are fracture healing and clinical outcome at 52 weeks after the start of treatment:

•Fracture healing will be scored by assessing union (union or non-union) and stability (stable or unstable).

o Union will be defined by evidence of bone trabeculae crossing the fracture site and absence of sclerotic borders adjacent to the fracture site, assessed using computed tomography (CT).

o Fracture stability will be assessed using upright cervical dynamic X-rays in lateral projection. A maximum of 2 mm movement at the fracture site is considered stable, over 2 mm movement at the fracture site is considered unstable [15].

•Clinical outcome; scored by calculation of the improvement compared to baseline in the Neck Disability Index (NDI) [16] score at 52 weeks after start of treatment. The NDI is a widely applied instrument to assess neck pain complaints. It was derived from the Oswestry index for back pain and the Pain Disability Index.

In the sample size calculation, the significance level was adjusted for the multiple primary outcome measures.

### Secondary outcome

Secondary clinical outcome parameters will be assessed by questionnaires to be completed by the patient. At baseline they will be completed at the hospital. During the follow-up period, they will be sent to the patient’s home. Radiological outcome parameters will be assessed by questionnaires to be completed by the treating surgeon and, in addition, again at the coordinating centre.

#### Clinical

•Myelopathy Disability Index (MDI) [17]: The MDI is a functional scoring system for cervical myelopathy that was originally designed for patients with rheumatoid arthritis. It consists of a selection of questions from the Standford Health Assessment Questionnaire.

•Visual Analogue Scale (VAS) for neck pain [18,19]: The VAS neck pain score indicates the intensity of experienced neck pain by drawing a mark on a 100 mm line. 0 mm symbolises ‘no pain’ , 100 mm symbolises ‘pain as worse as it could possibly be’.

•Short Form-36 (SF-36) [20,21]: The SF-36 is a generic health survey consisting of 36 questions. It consists of eight domains: physical functioning, physical restrictions, emotional restrictions, social functioning, somatic pain, general mental health, vitality and general health perception. It results in physical and mental health summary measures and a health utility index.

•EuroQol 5 Dimensions (EQ-5D) [22]: The EQ-5D is a tool to measure health outcome. It yields a descriptive profile and single index value for the patient’s health status.

•Type D Scale 14 (DS14) [23]: The DS14 is a standard assessment of negative affectivity, social inhibition, and Type D personality.

•Illness Perception Questionnaire-K (IPQ-K) [24]: The IPQ-K is a brief illness perception questionnaire, using a nine-item scale to rapidly assess the cognitive and emotional representations of the patient’s illness.

•Likert scale on recovery: The Likert scale used for this study is a seven-point scale on the patient’s perceived recovery from complaints and neck pain.

Not all of these questionnaires were already validated in the native languages of all countries involved in this trial. Therefore, prior to the start of the study they were validated by a process of bilateral translation. A native speaker of the target language who was also fluent in English first translated the questionnaire into the target language. A native speaker of English who also spoke the target language and was blinded to the original text then translated the text back into English. The original text and the back translated text were then compared at a consensus meeting. If the meaning of the two texts was agreed to be identical, the question was assumed to be adequately translated. Possible differences were discussed. More extensive options for transcultural adaptation of questionnaires were not considered feasible or necessary in the context of this trial.

#### Radiological

•Fracture displacement and direction: Displacement will be assessed by drawing lines along the posterior and lateral aspects of the odontoid process and the caudal body of C2. Displacement is expressed in mm. Direction is categorised as anterior, posterior, lateral, anterior-lateral or posterior-lateral.

•Grade of osteoporosis in C2: Osteoporosis will be classified according to the following criteria [4,25];

o None: normal trabecular pattern with normal cortical thickness.

o Mild: decrease in the amount of trabeculae with no areas of holes and normal cortical thickness.

o Moderate: absent trabeculae (holes) involving less than 25% of the transverse diameter of the bone with cortical thinning.

o Severe: absent trabeculae (holes) involving more than 50% of the transverse diameter of the bone with cortical thinning.

•Grade of degeneration in C0-C2 joint: Facet joint degeneration will be classified according to the following criteria [4,25];

o None: normal joint space with no osteophyte formation.

o Mild: narrowed joint space or normal joint space with osteophyte formation.

o Moderate: obliterated joint space with or without osteophyte formation.

o Severe: completely obliterated joint space, ankylosis or fusion of the joint.

#### General

•Complications: Complications resulting from the application of the different treatments will be monitored to identify potential differences in the occurrence or severity of complications between treatments.

•Re-interventions/Secondary surgery: One of the goals of the conservative treatment policy is to avoid surgery while still achieving fracture healing and a favourable clinical outcome. The rate of secondary surgery is hence an indication of the success or failure of this policy. Secondary interventions and the cause/motivation for its application will therefore be monitored.

### Sample size

Based on the recent literature review, the estimated possible difference in fracture union between the groups is 41% and in fracture stability this difference is 21% [12]. For both union and stability, however, a smaller difference of 20% would be clinically relevant and the study is powered to assess this difference. For the NDI, a 7.5 point difference (on a 50 point scale) is generally accepted as a minimal clinically important difference with a SD of approximately 10 in various psychometric studies [26-29]. Furthermore, it is expected that the number of patients that will be treated surgically will be twice the number of patients that are treated conservatively (2:1) [14]. Since three primary outcome hypotheses will be tested, the significance level (α) has to be divided by three. Based on the primary outcome parameters, the required sample size, assuming α = 0.05/3 = 0.0167 (two-sided) and β = 0.20 (80% power) and an expected drop out rate of 10%, is 275 for union, 198 for stability and 93 for the NDI. In conclusion, 275 patients will need to be recruited in order to give a reliable conclusion to the comparison of union, stability and clinical outcome between the surgically and conservatively treated groups.

### Statistical analysis

#### Descriptive statistics

Mean, median and standard deviations or median and ranges, if appropriate in case of skewed distributions, of descriptive parameters of primary and secondary outcomes will be reported.

#### Univariate analysis

Univariate analysis will be carried out using χ^2^-tests for dichotomised outcomes and T-tests for continuous outcomes. Intention-to-treat analysis will be used for cases that crossed over to other interventions.

•Primary analysis

The χ^2^-test will be used to test for differences between groups on union and stability at 52 weeks (α = 0.0167). T-test will be used to test for differences between groups on NDI improvement at 52 weeks (α = 0.0167).

•Secondary analysis

The χ^2^-test will be used to analyse the difference in dichotomised NDI. NDI will be dichotomised using the criterium of the minimal clinically important change (improvement) of 7.5 points out of 50. The relation between radiological parameters (union and stability) and NDI will be analysed by comparing the average NDI improvement for patients that acquired union/stability with those that did not.

#### Multivariate analysis

Multivariate analyses will be carried out using regression models with dichotomised union, stability and NDI as dependent variables, and with the secondary outcome parameters as independent variables and covariates. Propensity score analysis will be used to generate a model to predict the treatment received with the baseline variables:

•Patient age (both dichotomised (≤80 and >80 years) and continuous)

•Fracture characteristics (type of fracture)

•Fracture displacement (in mm and direction)

•Severity of osteoporosis in C2

•Facet joint degeneration in C1-C2

•NDI

•MDI

•VAS neck pain

•SF-36

•EQ-5D

### Withdrawal of individual subjects

Subjects can leave the study at any time for any reason if they wish to do so without consequences. As this is an observational study, this will only have influence on the study related assessments. Individual subjects withdrawn from the study will not be replaced. In the sample size a dropout rate of 10% was calculated for.

### Data analysis

Data analysis will be carried out based on the intention-to-treat principle. Cross-over-cases (e.g. surgery after failed conservative treatment) will be analysed among the original treatment group. This will not cause methodological problems because it is two healthcare strategies that are being compared, as opposed to two specific treatments. The reasons for cross-over of patients will be studied and reported. Causes of death will be analysed and a potential relation to the diagnosis and the applied treatment will be studied.

As-treated analysis will additionally be carried out in a sensitivity analysis and prognostic factors for the likeliness of surgery after failed conservative treatment will be studied.

## Discussion

In this article the rationale and design of a prospective cohort study on surgical versus conservative treatment for odontoid fractures in the elderly is described. There is a lacuna in evidence-based knowledge and guidelines in treating this patient population. To the authors’ knowledge, only one prospective study on this subject has yet been published, involving 159 patients ≥65 years with type II fractures but focusing just on characteristics associated with treatment success or failure and not on describing fracture healing [14]. However, the INNOVATE Trial will specifically aim to evaluate the outcome separately for patients younger and older than 80 years. Moreover, not only fracture union, but also fracture stability will be evaluated in the INNOVATE trial, which will be correlated to clinical outcome. A combination of (relative) fracture stability and favourable clinical outcome might be a legitimate endpoint of treatment in elderly patients, although long term effects have to be investigated. The objective of this trial is to identify which treatment strategy is most favourable for elderly patients with acute type II and III odontoid fractures and to identify factors that predict the success of either one of the available treatment in individual patients. The results of this trial will yield valuable information enabling more rational decision making in the treatment of odontoid fracture in elderly patients.

## Abbreviations

CRF: Case record form; CT: Computed tomography; NDI: Neck disability index; SIPS Group: Spine intervention prognostic study group; MDI: Myelopathy disability index; VAS: Visual analogue scale; SF-36: Short Form-36 Health Survey; EQ-5D: EuroQol 5 dimensions (generic measure of health); DS14: Type D Scale-14 (psychometric properties); IPQ-K: Illness perception questionnaire-K.

## Competing interests

The authors declare that they have no conflicts of interests.

## Authors’ contributions

JH is the project coordinator. WJ is responsible for the sample size calculation and design of the analysis. WP is the head of department and trial advisor. CV is the principal investigator and initiator. All authors contributed to the trial design and are responsible for the protocol. All authors read and approved the final manuscript.

## Pre-publication history

The pre-publication history for this paper can be accessed here:

http://www.biomedcentral.com/1471-2474/15/7/prepub

## References

[B1] Henaux PL Cueff F Diabira S Anterior screw fixation of type IIB odontoid fractures in octogenarians Eur Spine J 2011 21 2 235 239 10.1007/s00586-011-2044-7 PMC3265585 22008867

[B2] Hsu WK Anderson PA Odontoid fractures: update on management J Am Acad Orthop Surg 2010 18 7 383 394 20595131 10.5435/00124635-201007000-00001

[B3] Koech F Ackland HM Varma DK Williamson OD Malham GM Nonoperative management of type II odontoid fractures in the elderly Spine (Phila Pa 1976 ) 2008 33 26 2881 2886 10.1097/BRS.0b013e31818d5407 19092619

[B4] Osti M Philipp H Meusburger B Benedetto KP Analysis of failure following anterior screw fixation of Type II odontoid fractures in geriatric patients Eur Spine J 2011 20 11 1915 1920 10.1007/s00586-011-1890-7 21728075 PMC3207345

[B5] Platzer P Thalhammer G Oberleitner G Schuster R Vecsei V Gaebler C Surgical treatment of dens fractures in elderly patients J Bone Joint Surg Am 2007 89 8 1716 1722 10.2106/JBJS.F.00968 17671009

[B6] Tashjian RZ Majercik S Biffl WL Palumbo MA Cioffi WG Halo-vest immobilization increases early morbidity and mortality in elderly odontoid fractures J Trauma 2006 60 1 199 203 10.1097/01.ta.0000197426.72261.17 16456456

[B7] White AP Hashimoto R Norvell DC Vaccaro AR Morbidity and mortality related to odontoid fracture surgery in the elderly population Spine (Phila Pa 1976 ) 2010 35 9 Suppl S146 S157 20407346 10.1097/BRS.0b013e3181d830a4

[B8] Andersson S Rodrigues M Olerud C Odontoid fractures: high complication rate associated with anterior screw fixation in the elderly Eur Spine J 2000 9 1 56 59 10.1007/s005860050009 10766078 PMC3611343

[B9] Majercik S Tashjian RZ Biffl WL Harrington DT Cioffi WG Halo vest immobilization in the elderly: a death sentence? J Trauma 2005 59 2 350 356 10.1097/01.ta.0000174671.07664.7c 16294074

[B10] Reinhold M Bellabarba C Bransford R Radiographic analysis of type II odontoid fractures in a geriatric patient population: description and pathomechanism of the “Geier”-deformity Eur Spine J 2011 20 11 1928 1939 10.1007/s00586-011-1903-6 21796396 PMC3207349

[B11] Pal D Sell P Grevitt M Type II odontoid fractures in the elderly: an evidence-based narrative review of management Eur Spine J 2011 20 2 195 204 10.1007/s00586-010-1507-6 20835875 PMC3030710

[B12] Huybregts JG Jacobs WC Vleggeert-Lankamp CL The optimal treatment of type II and III odontoid fractures in the elderly: a systematic review Eur Spine J 2013 22 1 1 13 10.1007/s00586-012-2452-3 22941218 PMC3540294

[B13] Chapman J Smith JS Kopjar B The AOSpine North America geriatric odontoid fracture mortality study: a retrospective review of mortality outcomes for operative versus non-operative treatment in 322 patients with long-term follow-up Spine (Phila Pa 1976 ) 2013 38 13 1098 1104 10.1097/BRS.0b013e318286f0cf 23354104 PMC3678887

[B14] Fehlings MG Arun R Vaccaro AR Arnold PM Chapman JR Kopjar B Predictors of treatment outcomes in geriatric patients with odontoid fractures: AOSpine North America multi-centre prospective GOF study Spine (Phila Pa 1976) 2013 38 11 881 886 10.1097/BRS.0b013e31828314ee 23459135 PMC3678886

[B15] Knopp R Parker J Tashjian J Ganz W Defining radiographic criteria for flexion-extension studies of the cervical spine Ann Emerg Med 2001 38 1 31 35 10.1067/mem.2001.114319 11423809

[B16] Vernon H Mior S The neck disability index: a study of reliability and validity J Manipulative Physiol Ther 1991 14 7 409 415 1834753

[B17] Casey AT Bland JM Crockard HA Development of a functional scoring system for rheumatoid arthritis patients with cervical myelopathy Ann Rheum Dis 1996 55 12 901 906 10.1136/ard.55.12.901 9014584 PMC1010342

[B18] Huskisson EC Measurement of pain Lancet 1974 2 7889 1127 1131 4139420 10.1016/s0140-6736(74)90884-8

[B19] Carlsson AM Assessment of chronic pain. I. Aspects of the reliability and validity of the visual analogue scale Pain 1983 16 1 87 101 10.1016/0304-3959(83)90088-X 6602967

[B20] Ware JE Jr Sherbourne CD The MOS 36-item short-form health survey (SF-36). I. Conceptual framework and item selection Med Care 1992 30 6 473 483 10.1097/00005650-199206000-00002 1593914

[B21] Brazier JE Harper R Jones NM Validating the SF-36 health survey questionnaire: new outcome measure for primary care BMJ 1992 305 6846 160 164 10.1136/bmj.305.6846.160 1285753 PMC1883187

[B22] The EuroQol group EuroQol--a new facility for the measurement of health-related quality of life Health Policy 1990 16 3 199 208 10109801 10.1016/0168-8510(90)90421-9

[B23] Denollet J DS14: standard assessment of negative affectivity, social inhibition, and Type D personality Psychosom Med 2005 67 1 89 97 10.1097/01.psy.0000149256.81953.49 15673629

[B24] Broadbent E Petrie KJ Main J Weinman J The brief illness perception questionnaire J Psychosom Res 2006 60 6 631 637 10.1016/j.jpsychores.2005.10.020 16731240

[B25] Lakshmanan P Jones A Howes J Lyons K CT evaluation of the pattern of odontoid fractures in the elderly–relationship to upper cervical spine osteoarthritis Eur Spine J 2005 14 1 78 83 10.1007/s00586-004-0743-z 15723251 PMC3476682

[B26] Carreon LY Glassman SD Campbell MJ Anderson PA Neck disability index, short form-36 physical component summary, and pain scales for neck and arm pain: the minimum clinically important difference and substantial clinical benefit after cervical spine fusion Spine J 2010 10 6 469 474 10.1016/j.spinee.2010.02.007 20359958

[B27] Cleland JA Fritz JM Whitman JM Palmer JA The reliability and construct validity of the neck disability index and patient specific functional scale in patients with cervical radiculopathy Spine (Phila Pa 1976 ) 2006 31 5 598 602 10.1097/01.brs.0000201241.90914.22 16508559

[B28] Young BA Walker MJ Strunce JB Boyles RE Whitman JM Childs JD Responsiveness of the neck disability index in patients with mechanical neck disorders Spine J 2009 9 10 802 808 10.1016/j.spinee.2009.06.002 19632904

[B29] Young IA Cleland JA Michener LA Brown C Reliability, construct validity, and responsiveness of the neck disability index, patient-specific functional scale, and numeric pain rating scale in patients with cervical radiculopathy Am J Phys Med Rehabil 2010 89 10 831 839 10.1097/PHM.0b013e3181ec98e6 20657263

